# A Sitewise Model of Natural Selection on Individual Antibodies via a Transformer–Encoder

**DOI:** 10.1093/molbev/msaf186

**Published:** 2025-08-05

**Authors:** Frederick A Matsen, Kevin Sung, Mackenzie M Johnson, Will Dumm, David Rich, Tyler N Starr, Yun S Song, Philip Bradley, Julia Fukuyama, Hugh K Haddox

**Affiliations:** Computational Biology Program, Fred Hutchinson Cancer Center, Seattle, WA 98109, USA; Department of Genome Sciences, University of Washington, Seattle, WA 98195, USA; Department of Statistics, University of Washington, Seattle, WA 98195, USA; Howard Hughes Medical Institute, Seattle, WA 98109, USA; Computational Biology Program, Fred Hutchinson Cancer Center, Seattle, WA 98109, USA; Computational Biology Program, Fred Hutchinson Cancer Center, Seattle, WA 98109, USA; Computational Biology Program, Fred Hutchinson Cancer Center, Seattle, WA 98109, USA; Computational Biology Program, Fred Hutchinson Cancer Center, Seattle, WA 98109, USA; Department of Biochemistry, University of Utah, Salt Lake City, UT 84112, USA; Computer Science Division and Department of Statistics, University of California, Berkeley, CA 94720, USA; Computational Biology Program, Fred Hutchinson Cancer Center, Seattle, WA 98109, USA; Institute for Protein Design, Department of Biochemistry, University of Washington, Seattle, WA 98195, USA; Department of Statistics, Indiana University, Bloomington, IN 47405, USA; Computational Biology Program, Fred Hutchinson Cancer Center, Seattle, WA 98109, USA

**Keywords:** antibody, B cell receptor, affinity maturation, natural selection, transformer architecture

## Abstract

During affinity maturation, antibodies are selected for their ability to fold and to bind a target antigen between rounds of somatic hypermutation. Previous studies have identified patterns of selection in antibodies using B cell repertoire sequencing data. However, these studies are constrained by needing to group many sequences or sites to make aggregate predictions. In this paper, we develop a transformer–encoder selection model of maximum resolution: given a single antibody sequence, it predicts the strength of selection on each amino acid site. Specifically, the model predicts for each site whether evolution will be slower than expected relative to a model of the neutral mutation process (purifying selection) or faster than expected (diversifying selection). We show that the model does an excellent job of modeling the process of natural selection on held out data, and does not need to be enormous or trained on vast amounts of data to perform well. The patterns of purifying vs diversifying natural selection do not neatly partition into the complementarity-determining vs framework regions: for example, there are many sites in framework that experience strong diversifying selection. There is a weak correlation between selection factors and solvent accessibility. When considering evolutionary shifts down a tree of antibody evolution, affinity maturation generally shifts sites towards purifying natural selection, however this effect depends on the region, with the biggest shifts toward purifying selection happening in the third complementarity-determining region. We observe distinct evolution between gene families but a limited relationship between germline diversity and selection strength.

## Introduction

Antibodies are a miracle of adaptive immunity, capable of binding a huge diversity of targets. They are generated as B-cell receptors (BCRs) via random V(D)J recombination, then encounter positive and negative selection in the bone marrow. After antigen stimulation, they develop via affinity maturation, in which they encounter high rates of mutation and a process of natural selection in the germinal center. Studies have used high-throughput sequencing to read out the results of this process for hundreds of thousands to millions of antibody sequences from dozens of human individuals. How can we use these natural data to inform our understanding of affinity maturation, as well as strategies for designing vaccines and therapeutics?

There has been an explosion of new language models for antibodies (Graves et al. 2020; Ruffolo et al. 2021; Bachas et al. 2022; Leem et al. 2022; Olsen et al. 2022; Hie et al. 2024; Wang et al. 2023; Barton et al. 2024; Jing et al. 2024; Kenlay et al. 2024; Ng and Briney 2025; Olsen et al. 2024; Shanker et al. 2024; Turnbull et al. 2024; Wang et al. 2025). These models have used a masked language objective: namely, the model is asked to predict a subset of the sequence that has been masked (i.e. hidden) away. Although this procedure has proven very effective in modeling of human language, it does not take into account a key difference between human language and the language of antibodies: antibodies do not appear completely *de novo*, but rather as recombinants and mutational descendants of known sets of germline genes. In contrast, human language has only grammatical and semantic constraints. As a result, masked-language models of antibodies can achieve high predictive accuracy merely because they are adept at predicting germline-encoded residues, rather than patterns of affinity maturation (Ng and Briney 2025; Olsen et al. 2024). One can modify the masked-language objective by using a focal loss function that down-weights the loss of well-predicted labels (Olsen et al. 2024), or focusing masking on nontemplated regions (Ng and Briney 2025). This is important progress. However, there are additional factors that shape the observed set of antibody sequences in addition to germline-encoded residues.

Indeed, variation in antibody sequence is strongly constrained by patterns of somatic hypermutation (SHM). Numerous studies have shown the strength of context-sensitive biases in somatic mutation (Yaari et al. 2013; Elhanati et al. 2015; Cui et al. 2016; Feng et al. 2019) and others have shown that these biases strongly shape affinity maturation (Uduman et al. 2011; Yaari et al. 2012; McCoy et al. 2015; Sheng et al. 2017). In fact, we have recently shown that a *neutral* SHM model trained on out-of-frame antibody sequences outperforms antibody language models on the task of predicting the course of affinity maturation (Johnson et al. 2025 ), echoing previous studies showing that many substitutions in the course of affinity maturation are affinity-neutral (Murugan et al. 2018). For this reason, one cannot simply take the empirical distribution of observed amino acids as a direct readout of the suitability of those amino acids for that site: this conflation of two effects limits the effectiveness and interpretability of antibody language models.

In this paper, we take a first step towards resolving these issues by using neural networks to separately model SHM and selection during affinity maturation in humans. We leverage the fact that substitutions in “out-of-frame” antibody sequences arise via SHM, while those in “in-frame” antibody sequences arise via both SHM and selection. Previously, we trained a neural network on out-of-frame sequences to predict rates of SHM at each site of an input antibody sequence (Sung et al. 2025). Here, we train a separate neural network on in-frame sequences to predict whether nonsynonymous substitutions at each site of an input antibody sequence occur more or less frequently than expected under the SHM model. Thus, if we assume that the first model accurately captures SHM, the second model will only learn the effects of selection. We call this model a deep natural selection model (DNSM).

We find that the model has excellent out-of-sample model fit, and that it does not need to be especially large or be trained on huge data sets to perform well. Our analysis reveals distinct patterns of natural selection across antibody regions, with a surprising prevalence of diversifying selection in framework regions (FWRs), strong purifying selection at conserved and electrostatic interaction sites, and a weak correlation with solvent accessibility. Our code is open source and at https://github.com/matsengrp/netam. The reproducible experiments, including trained model weights, are available at https://github.com/matsengrp/dnsm-experiments-mbe. We have made interactive 3D visualizations of the natural selection of the human antibodies in the SAbDAb database (Dunbar et al. 2014) using dms-viz (Hannon and Bloom 2024) available at https://matsen.group/dnsm-viz/v1/.

## Results

### Methods Overview

Here we present a brief overview of the goals and methods of the project. For details, please refer to the *Materials and methods* section.

Our high-level goal is to train a model that deconvolves SHM and selection in human antibody evolution at the level of individual sites and sequences. The SHM component of the model is a small neural network (about 6,000 parameters) with separate embedding layers and 1D convolutions for rate and substitution prediction, each followed by their respective linear heads; it is trained on nonfunctional out-of-frame sequences (Sung et al. 2025) and fixed for the purposes of this paper. The selection component of the model (the DNSM) is trained on evolutionary trees of functional in-frame antibody sequences to predict whether nonsynonymous substitutions occur more or less frequently than expected under the SHM model.

**Fig. 1.**
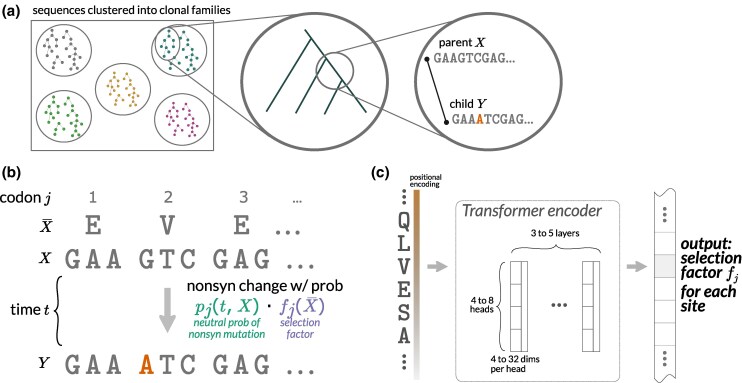
The goal of the method is to infer a mapping *f* from an amino acid sequence $\bar{X}$ to a *selection factor* for each site *j*. This is a multiplier of the neutral probability of nonsynonymous substitution: sites undergoing diversifying selection will have a selection factor >1, while those with purifying selection will have a selection factor <1. a) The data consists of pairs of sequences, one “parent” codon sequence *X* and one “child” codon sequence *Y*, inferred from clonal families. b) The inference of the model involves fitting *f* across many parent–child pairs, jointly with a branch length *t* for each pair. The neutral probability model $p_{j}(t,X)$ is inferred separately using out-of-frame data. The goal is to predict the probability of nonsynonymous substitution at every site of each parent–child pair. c) The mapping *f* is parameterized using a transformer–encoder neural network of relatively modest size. Further detail is provided in supplementary fig. S1, Supplementary Material online.

Specifically, the raw training data consist of antibody sequences from past studies that deeply sequenced human antibody repertoires, described more below. For each repertoire, we clustered sequences into clonal families (Fig. 1a). Then, for each family, we reconstructed a phylogenetic tree of the sequences, using ancestral sequence reconstruction to infer sequences at internal nodes (Spisak et al. 2020). These were reconstructed once and fixed.

We trained the model to predict evolution along the branches of these trees using the following strategy. For each pair of nodes directly connected by a branch, which we refer to as “parent–child pairs” (PCPs; Fig. 1a), we fed the model the parent node’s DNA sequence and trained it to predict the probability of observing nonsynonymous substitutions at each site of the child node’s sequence. Specifically, for each codon site *j* in a sequence, the model computes this probability as the product of two variables: $p_{j}$ and $f_{j}$ (Fig. 1b). The first variable, $p_{j}$, is the probability of observing a nonsynonymous substitution at that site under neutrality, given a branch length of time *t* (see Methods for details). This probability is computed by the SHM component of the model, and assumes an independent Poisson mutation process at each of the three nucleotide positions in a given codon. The second variable, $f_{j}$, is a *selection factor* that scales $p_{j}$ to account for the effect of selection on the substitution probability. This selection factor is computed by the DNSM, and has an interpretation similar to a dN/dS ratio (Yang and Bielawski 2000), in that values greater than 1 imply diversifying selection, values less than 1 imply purifying selection, and 0 is the strongest possible purifying selection.

A key aspect of the model is that the predicted site-specific selection factors depend on the input parent amino-acid sequence, not just the position of a given site in the protein. As we show below, this allows the model to learn how selection at a site depends on the surrounding sequence context.

We parameterize the model using a shared amino-acid embedding followed by a transformer–encoder (Vaswani et al. 2017) neural network followed by a simple linear layer (Fig. 1c; see Methods) and a “wiggle” activation function. These three components are trained simultaneously. Although we tested a variety of model sizes (supplementary table S2, Supplementary Material online), we will use the “1.2 M” model for the manuscript unless stated otherwise. This model has 8 heads, 16 dimensions per head, 5 layers, and 1,192,449 parameters.

We used almost 750,000 PCPs derived from 25 subjects (Vergani et al. 2017; Tang et al. 2020; Jaffe et al. 2022) for training/validation, and used over 21,000 PCPs from 50 subjects from (Rodriguez et al. 2023) for testing data (see supplementary table S1, Supplementary Material online for details and statistics about the data). Unless otherwise stated, we used the Jaffe+Tang (Vergani et al. 2017; Tang et al. 2020; Jaffe et al. 2022) data for training/validation and the Rodriguez data for testing. We jointly optimized model parameters and PCP branch lengths, alternating between the two.

### Deep Selection Models Accurately Predict Substitution Probability

First, we tested the model’s ability to predict substitution probabilities in PCPs in the Rodriguez test dataset (Rodriguez et al. 2023) not used for training. In fact, this data set was generated using a different sequencing methodology than that for the training data. For each site in the parent of each PCP, we computed the probability of having an amino-acid substitution at that site in the corresponding child. We then grouped together all PCPs within the same V-gene families, aligned their sites according to the IMGT scheme, and then totaled the substitution probabilities at each site to obtain an estimate of the number of substitutions we expect to see at each site across all PCPs in the group. We then compared this to the actual number of substitutions that were observed at each site in the PCPs. If the observed and expected counts match, then the model is doing a good job, on average, of predicting site-specific probabilities of substitution.

**Fig. 2.**
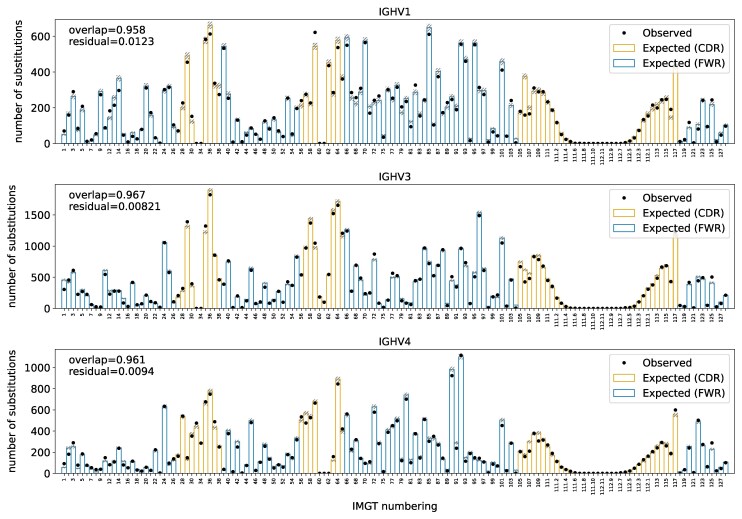
Model fit for predicting the per-site probability of nonsynonymous substitution for a dataset from the Rodriguez dataset (Rodriguez et al. 2023), partitioned by V-gene family. Points show observations and bars show predictions; the gray hatched segments of the bars show standard deviation on the predicted number of substitutions. Sites aligned according to the IMGT numbering scheme (Lefranc 1997). Compare model fit for a baseline model with a single selection factor (supplementary fig. S2, Supplementary Material online) and model fit for a purely neutral model (supplementary fig. S3, Supplementary Material online).

We found that the model had excellent performance in this task (Fig. 2). We assessed fit using an “overlap” metric, which quantifies the size of the intersection of the histograms divided by the average area of the histograms. For the DNSM model combined with the model of SHM, this metric is around 0.96. In comparison, if we train a baseline model that only includes a single selection factor for modeling all sites, the overlap decreases to less than 0.8 (supplementary fig. S2, Supplementary Material online); a neutral model has an overlap of slightly less (supplementary fig. S3, Supplementary Material online). This single-selection-factor model is analogous to repertoire-wide models of selection in which every sequence is assumed to be under the same selection pressure (Hoehn et al. 2019).

These results establish that we can make accurate predictions of the per-site probability of nonsynonymous substitution when aggregated by gene family. In fact, comparing overlap values in a recent benchmarking paper (Johnson et al. 2025), the DNSM model with the SHM model performs much better than protein language models, including antibody-specific ones. The DNSM performance is even better than a custom framework modeling the evolution of a single naive ancestor diversifying to fit a corresponding single antigen, via a bespoke somatic hypermutation model using the naive sequence used in the experiment, and a deep mutational scan for that antibody-antigen pair (DeWitt et al. 2025).

### Model Performance Improves Modestly with Data and Model Size

Previous deep-learning antibody models have made significant gains by increasing the size of models and data sets (Barton et al. 2024). We wished to understand how scaling worked for our models. Thus, we performed a series of model fits using models of different sizes (details in supplementary table S2, Supplementary Material online), which we label according to their abbreviated number of parameters. For each size, we trained the models with three different data sets: Tang50k (an evenly-spaced 50,000 PCP subset of the Tang et al. (2022) data), Tang (the entire (Tang et al. 2022) data) and then Jaffe+Tang (these data combined with the 10X data of Jaffe et al. 2022). By “evenly-spaced” we mean that we skipped evenly through the PCP file to get a representative sample of the data. Because the PCP file is organized by clonal family, this reduces the number of PCPs from the same clonal family in the training set. We then tested the models on the Rodriguez test dataset (Rodriguez et al. 2023), as before.

**Fig. 3.**
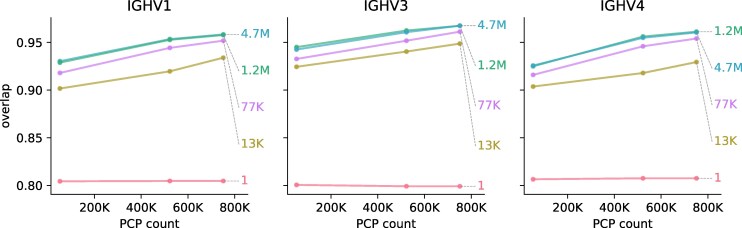
Model performance by model size and training-set size. Each line represents one model size, and is labeled by the number of parameters of that model, with more detail about model structure in supplementary table S2, Supplementary Material online. Each panel shows the overlap on the Rodriguez test dataset (Rodriguez et al. 2023) stratified into gene families. The *x* axis is the dataset size, which has three points: a 50 K subset of the Tang et al. (2022) data, the entire (Tang et al. 2022) data, and then these data combined with the 10X data of Jaffe et al. (2022).

Our models showed modest gains when increasing model size and training data volume (Fig. 3). Indeed, our best model for both in-sample data and out-of-sample data had 1.2 million parameters, and increasing the model size further did not improve performance; reducing it to 77 thousand parameters only slightly reduced performance. Furthermore, increasing data volume only modestly increased performance.

### Deep Selection Models can be Trained with Around a Half-Million PCPs

With any large model there is potential for overfitting, and in this context, one may be concerned that around a million parameters is too many to train with the data that we use. To investigate this issue, we started with a selection model trained with Jaffe+Tang data as above, which we took to be a ground-truth model. We used this model along with the corresponding model of SHM to simulate sequences down the trees to get a “mock” version of the training data. We made sure the mutation positions in the simulated data precisely matched the expected positions under the ground-truth model, demonstrating that the simulation worked as expected (overlap ≈0.996; supplementary fig. S4, Supplementary Material online). We then used these sequences to retrain a DNSM model which we could use to compare selection factor predictions on the Rodriguez test dataset.

**Fig. 4.**
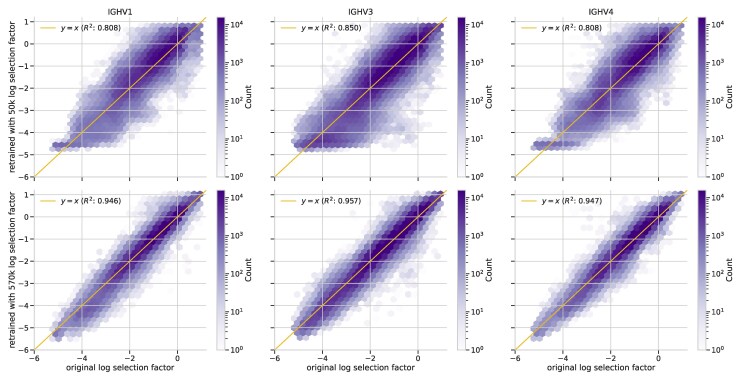
Selection factors for a model fit on simulated data agree well with selection factors used to simulate the data on a panel of test sequences. Each point in this hexbin plot is one site of one sequence in one of the test sequences. The *x* axis position is the selection factor predicted for that site using the model used to simulate the sequences, while the *y* axis position is the selection factor predicted using a model that was fit on the designated amount of data. Note that the count in the hexbin plot is on the log scale. For additional training sizes see supplementary fig. S5, Supplementary Material online. For a version of this plot with selection factors rather than log selection factors see supplementary fig. S6, Supplementary Material online.

We found excellent prediction of selection factors when training with around a half million simulated PCPs. Specifically, when comparing the refit-model-prediction for every site of every sequence in the testing data to the corresponding ground-truth-model prediction, we found a coefficient of determination ($R^{2}$) of around 0.95 when compared against perfect agreement (the y=x line) on the log-log scale (Fig. 4). When training with an evenly-spaced sample of only 50 thousand PCPs from this simulated data, the correlation was significantly worse. Thus we believe that the data volume that we have, we are able to recover parameters accurately.

### Natural Selection Varies Substantially between Sites and does not Partition Neatly into CDR vs FWRs

Antibodies are traditionally divided into two regions: the complementarity-determining regions (CDRs) and the intervening FWRs. The CDRs are the regions that contact antigen, and as such are conventionally thought to be the regions under diversifying selection, while the FWRs are thought to be under purifying selection to maintain the structure of the antibody. Our models allow us to test this conventional wisdom in detail. To do so, we used the model to predict a selection factor for every site of every human antibody in the Rodriguez (Rodriguez et al. 2023) dataset, and aligned the results according to the IMGT scheme.

**Fig. 5.**
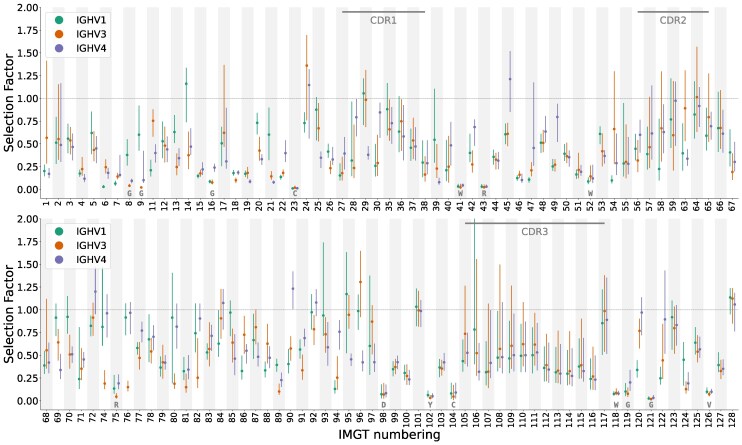
Overall patterns of natural selection for the human antibodies in the Rodriguez (Rodriguez et al. 2023) dataset, aligned via the IMGT numbering scheme. These sequences were partitioned into IGHV gene family, and for every site the median (point) and interquartile range (line) are shown. Only sites present in at least one sequence for each gene family are shown. Consensus amino acids are shown in gray monospace font for the sites with the 15 smallest selection factors. CDR regions are marked, and the *k*th FWR regions appear just before the *k*th CDR region.

We found that predicted selection factors were highly variable between sites (Fig. 5). At several sites, the predictions were close to zero for nearly all antibodies, indicating strong purifying selection at these sites. Many of these sites match ones that have been previously identified as being structurally important, including highly conserved C-W motifs that serve as landmarks for identifying the CDR1 and CDR3 loops, as well as nearby glycine residues (group of Andrew C R Martin 2024). We note that the model was not provided with a structural alignment, thus the transformer itself learned to align the W marking at the end of the CDR3. The sites under strong purifying selection also include a RRDY motif (sites 43, 75, 98, 102) consisting of amino acids that make electrostatic interactions at the base of the heavy-chain variable loop (supplementary fig. S7, Supplementary Material online). This has been called a “charge cluster” by (Honegger et al. 2009); others have identified low substitution frequency at these sites (Kirik et al. 2017).

We found that patterns of diversifying selection did not neatly partition into CDR vs. framework regions. We expected that sites in the CDR loops would tend to have higher selection factors than sites in the FWRs, since the CDR loops often make direct contact with antigens, and since affinity-enhancing substitutions often occur in these loops for that reason. Indeed, many sites in CDR loops had high predicted selection factors, some reaching values greater than 1.0, indicating that selection does tend to be more diversifying at these sites. However, many sites in the FWR also had diversifying selection.

For example, IMGT site 24 is the only site with a median selection factor greater than one in two V gene families, and it is a FWR site. This site falls directly after the first conserved cysteine but is well before the start of the CDR1 loop, still forming part of a beta sheet (an example visualization can be found at https://matsen.group/dnsm-viz/v1/?pdbid=3b2v; note that due to IMGT numbering this is the 23rd site of the sequence). Because this seemed like a surprising result, we verified the surprisingly high selection factors at site 24 using a simple counting-based approach. This site indeed showed a strong excess of nonsynonymous substitutions, and furthermore, this excess cannot be explained by SHM biases (supplementary fig. S8, Supplementary Material online).

Finally, the median selection factors for sites in the CDR3 region were not as high as we might have expected. On the other hand, they did have very wide interquartile ranges, indicating that there is substantial variation in selection factors between antibodies. We explore this in the following two sections, showing that the CDR3 has strikingly different patterns of selection between naive and mature antibodies, and that evolutionary dynamics also depend on CDR3 length.

### Natural Selection Estimates Change as Antibodies Evolve

We wished to investigate how natural selection changes as BCRs evolve. The ability to perform such an analysis is a unique feature of our model, which makes predictions for a single sequence rather than aggregate predictions for a multiple sequence alignment. To do so, we used a model trained only on the Tang (Tang et al. 2020) data, evaluated on clonal families of at least size 10 in the Jaffe (Jaffe et al. 2022) data. We did so because this analysis required large and high-accuracy clonal families, which are more reliably inferred using paired heavy and light clustering. For each clonal-family phylogenetic tree, we found the node closest to the root (in terms of total branch length; excluding the inferred naive sequence), which we called the “ancestral” node, and the node farthest from the root, which we called the “derived” node, and examined how predicted selection factors differed between these nodes.

**Fig. 6.**
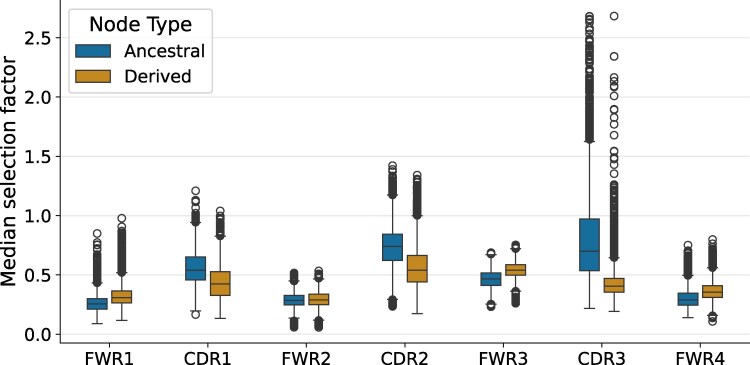
Median selection factor estimates on the Jaffe data per region across clonal families for “ancestral” nodes closest to the root and “derived” nodes that are farthest from the root. Results are shown for a model trained only on the Tang data.

In general, selection factors in the CDRs tended to be substantially lower in derived nodes compared to ancestral nodes, while selection factors in FWRs tended to be slightly higher in the derived nodes (Fig. 6). Thus, the model predictions indicate that there are increased levels of purifying selection in CDRs as antibodies mature. Indeed, it makes intuitive sense that sites in CDRs would tend to be under more diversifying selection early in the affinity-maturation process, and shift to more purifying selection after having acquired affinity-enhancing substitutions.

We also note that the selection factors in the FWRs were not uniformly low for either ancestral or derived nodes (Fig. 6). Indeed, of the three FWRs, FWR3 had the most sites with high selection factors, and the median selection factor in framework 3 was actually comparable with the median selection factor in CDR1 (the CDR region with the lowest selection factors). The FWR3 is different than the other FWRs, being much longer and including a loop (IMGT 80–87) that has been implicated in antigen binding, which has been called the fourth CDR (Kelow et al. 2020). Overall, the diversity of selection factors in FWRs goes against the classical view that these regions merely serve as a fixed structural scaffold that evolves under strong purifying selection.

### Selection Differs between CDR3s of Different Lengths

**Fig. 7.**
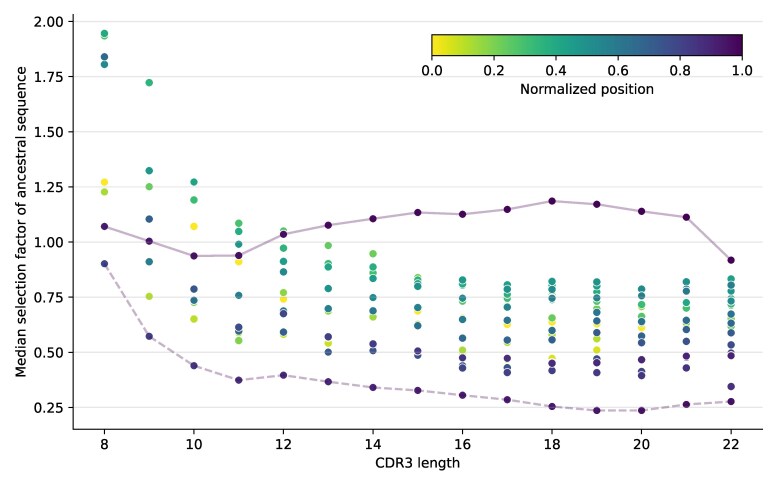
The median selection factor for each site across ancestral sequences for the Jaffe (Jaffe et al. 2022) data partitioned by CDR3 length. Each point represents a site in a CDR3 sequence of a given length, and is colored according to the “normalized position”: the position divided by CDR3 length. The points for the last site of the CDR3 are connected with a solid line, and the points for the second-to-last site (overwhelmingly aspartic acid) are connected by a dashed line. This is a compressed version of the results, which may be easier to understand after viewing supplementary fig. S9, Supplementary Material online.

We also found that selection factors in CDR3 depended on this region’s length. To isolate this effect from the one described in the previous section, we specifically examined the effect of length in context of ancestral sequences. When we aggregated per-site selection factors for ancestral sequences with the same CDR3 length, we found that sites in shorter CDR3s tend to have higher selection factors (Fig. 7, supplementary fig. S9, Supplementary Material online). Specifically, very short CDR3s are predicted to have primarily diversifying selection acting on their CDR3s, while for longer CDR3s most sites are predicted to have purifying selection. The exception to this was the last site in CDR3, which has a median selection factor of ∼1 regardless of length.

One explanation for this finding is that only a few sites in a given CDR loop are usually under diversifying selection, such that the median selection factor tends to go down as loop length increases. On the other hand, we found that the amino acid content of the ancestral CDR3s differs between short and long CDR3s (supplementary fig. S9, Supplementary Material online), which may also explain the difference.

The aspartic acid (the D in supplementary fig. S9, Supplementary Material online) forming the second to last amino acid of the CDR3 is consistently under strong purifying selection (dashed line in Fig. 7). Why? By inspecting structures, we have found that this residue often makes contact with amino acids at the beginning of the CDR3, such as making a salt bridge with the CAR motif at the beginning of the CDR3. These salt bridges have been observed previously (Chien et al. 1989), and the strong selection observed here vouches for their importance.

### Germline Amino-acid Diversity Does Not Correlate with Aggregate Selection Factors Except at known Conserved Residues

We wished to understand if there was a connection between per-site diversity of germline amino acids and their natural selection. As an obvious case of this connection, the cysteine-tryptophan residues bounding the CDRH1 and CDRH3 are identical between V genes and also highly conserved in the course of BCR evolution. However, one could imagine that a more subtle connection would be present. If there is a correlation, that means that the deep-time evolution is paralleling the affinity maturation process, and points to similarities in selective pressures. A lack of correlation could indicate differences in selection. Previous analyses have found a strong correlation (Schwartz and Hershberg 2013).

To investigate this question, we calculated germline entropy and selection factor on the Rodriguez (Rodriguez et al. 2023) data, where sites are aligned using the IMGT numbering. Germline Shannon entropy is calculated using counts weighted by their presence in the data set, with the corresponding sequence logo in the bottom panel.

**Fig. 8.**
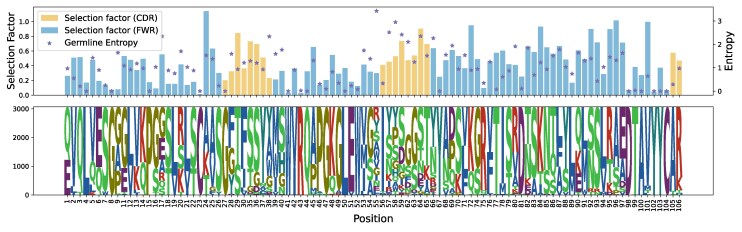
A per-site comparison of germline entropy and selection factor on the Rodriguez (Rodriguez et al. 2023) data. Germline Shannon entropy is calculated using counts weighted by their presence in the data set, with the corresponding sequence logo in the bottom panel. Selection factor is the median per-site selection factor for the dataset. Sites aligned using the IMGT numbering.

We found a weak correlation between the two measures (Fig. 8). The strongest effect appeared to be that sites with very low entropy also had low selection factors (supplementary fig. S13, Supplementary Material online). Indeed, when we removed sites with very low entropy (less than 0.2), the correlation between entropy and selection factor dropped from 0.15 to 0.03. Thus, our conclusion is that germline amino-acid diversity does not correlate with aggregate selection factors except via known conserved residues.

### Selection Factors Weakly Correlate with Solvent Accessibility

We next sought to investigate selection factors in context of antibody structures. In general, in protein evolution, a residue’s evolutionary rate positively correlates with its level of solvent exposure, where residues that are more exposed tend to evolve faster than residues that are more buried (Franzosa and Xia 2009). Thus, we expected that a site’s solvent exposure would help explain the high variability in selection factors between antibody sites observed above. To test this hypothesis, for each human antibody in the SAbDAb database (Dunbar et al. 2014), we used the antibody’s sequence to predict each site’s selection factor and used the antibody’s structure to calculate each residue’s relative solvent accessibility (RSA; the antigen component of the structure was removed from the RSA calculation).

**Fig. 9.**
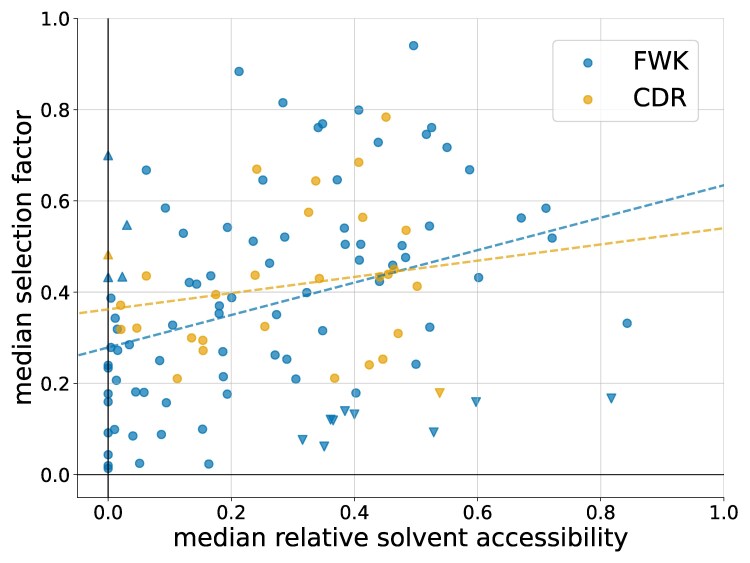
A per-IMGT-site comparison of median relative solvent accessibility and median selection factor on the SAbDAb. Triangles pointing down are exposed sites with relatively low selection factor, namely the 10 sites with the lowest selection factors that have relative solvent accessibility greater than 0.3. Triangles pointing up are buried sites with relatively high selection factor, namely the 5 sites with the highest selection factors that have relative solvent accessibility less than 0.05.

As expected, we observed a positive correlation between selection factor and RSA, but the correlation was weak (Fig. 9). We found a Pearson correlation coefficient *r* between the two variables across sites of 0.186 in the FWK region and 0.363 in the CDR region (p less than machine tolerance).

In order to understand this finding, we examined sites with surprisingly high or low selection factors given their level of RSA. First, we examined exposed sites predicted to be under purifying selection, namely sites with high RSA (greater than 0.3) but low selection factors (triangles pointing down in Fig. 9; supplementary table S3, Supplementary Material online). Despite being relatively exposed, the low selection factors indicate that these sites are structurally or functionally important. This set of sites includes W118, which is part of the highly conserved C-W motif bounding the CDR3 loop. It also includes several glycine residues, many of which occur in loops, a context in which glycine can be important for enabling backbone flexibility. To validate the predicted purifying selection at these sites, we performed a counting analysis of synonymous versus nonsynonymous mutations as before for the four nontryptophan sites with the lowest selection factor among the exposed sites, and indeed found a clear excess of synonymous mutations (supplementary fig. S10, Supplementary Material online). Tryptophan (W) encoding sites were not included in this analysis because they only have one codon and thus no synonymous mutations.

Next, we examined buried sites, namely sites with low RSA (less than 0.05), yet relatively high selection factors (triangles pointing up in Fig. 9; supplementary table S4, Supplementary Material online). Despite being buried, the relatively high selection factors indicate that these sites are at least somewhat tolerant of nonsynonymous substitutions. Again, to validate this prediction, we performed a counting analysis and found a substantial number of both synonymous and nonsynonymous mutations (recall that because these selection factors are in the 0.4 to 0.7 range, we expect a mix of both mutation types). The most frequent mutation for the three buried sites with the highest selection factors were to valine: L to V, I to V, and A to V (supplementary fig. S11, Supplementary Material online). These are relatively conservative changes. However, radical changes between amino acids with very different properties such as L to R, I to S, and A to E were not observed. This case study underlines how the DNSM predicts an aggregate selection factor, and just because the model predicts (correctly in this case) that amino acid substitutions are tolerated at this site does not mean that any amino acid will suit.

Overall, we found that selection factors describe more than just solvent accessibility, and aren’t obvious from structure alone.

### Overall Rates of Somatic Hypermutation are Lower at Sites under Strong Purifying Selection

Previously, it has been shown that the SHM process focuses mutations in the CDRs (Shapiro et al. 2002; Saini and Hershberg 2015; Vieira et al. 2018). Sites in CDRs tend to evolve under more diversifying selection. This raises an interesting hypothesis: that rates of SHM are higher at sites that evolve under more diversifying selection and lower at sites that evolve under more purifying selection. To investigate this hypothesis, we separately considered the aggregated mutability and selection for the V3 and V4 gene families. Mutability and selection are indeed correlated for these gene families (supplementary fig. S12, Supplementary Material online), with strong signal that sites with low selection factors also have low mutability. Although the correlation is not perfect, it is still striking that a correlation exists between the mutation process, which happens independent of antibody function, and the affinity selection process.

## Discussion

In this paper, we developed a transformer–encoder to predict per-site selection strength on individual antibody sequences. The resulting selection factors describe the fate of mutations to a parent sequence in the germinal center. Specifically, they describe if a mutation is more (when the selection factor is greater than one) or less (when the selection factor is less than one) likely to survive natural selection in the germinal center relative to a baseline rate of somatic hypermutation. The model is unique among models to predict natural selection in that it delivers a per-sequence-per-site prediction.

This approach builds on a foundation of evolutionary modeling of antibodies using coarser groupings: either grouping sequences to get per-site resolution, or grouping sites to get finer resolution in terms of sequences. For example, one vein of work has been to partition antibody sequences into CDR vs framework and compare aggregate evolution between them (Yaari et al. 2012; Nourmohammad et al. 2019), including separating sequences by location in evolutionary trees (Yaari et al. 2015). Our study shows that natural selection pressures vary significantly between sites of a given region. Another approach is to partition by germline genes and consider per-germline-gene evolution per site (McCoy et al. 2015 ; Yaari et al. 2015; Kirik et al. 2017; Sheng et al. 2017). However, this misses opportunities to gather strength from similar germline genes. It also does not provide selection estimates for sites that are not germline-encoded or hard to align to germline, namely mutated residues or the CDR3. In our study, we observed interesting patterns in the CDR3 before and after a molecular substitution, and when partitioning by CDR3 length.

From the perspective of language modeling, our study is distinguished from other studies by using an explicitly evolutionary loss function. Recent studies have moved beyond a simple missing-token prediction task to a task incorporating germline gene or basic structural knowledge (Ng and Briney 2025; Olsen et al. 2024). We take this approach to its logical extent by conditioning on the complete parent sequence and predicting the probability of a nonsynonymous substitution.

From the perspective of evolutionary modeling, our study is distinguished from previous studies by the detail of the mutation and selection models. Classical models of natural selection fit mutation and selection parameters simultaneously on same sequences, using synonymous mutations to inform the mutation side (Yang and Bielawski 2000). Here we used a complex mutation model trained on out-of-frame data, and then train a selection model on in-frame data. We used a transformer–encoder network to be able to express complex patterns of selection for individual sequences. We believe that a transformer–encoder is an appropriate tool to express evolutionary patterns on the high diversity of antibody sequences coming from many germline genes, and which can recombine in various ways with insertions and deletions. It’s difficult to imagine a simpler model that would be up to this task.

Our central scientific conclusion is that there are intricate overall patterns of natural selection in antibodies. These patterns are not specific for a given antigen, but rather are shaped by the current mix of pathogens to which the study subjects have been exposed. One could imagine a world in which the germline genes, recombination, and mutation process has evolved to be optimally poised for affinity maturation to this mix; in such a world there would be no overall selection patterns. However, in repertoire data, we did indeed see such overall patterns across sites, and predicted per-site selection factors differ between sequences in a complex way.

Purifying and diversifying natural selection do not clearly partition into the framework and CDR regions, although many patterns of purifying natural selection have a structural interpretation. There are germline-encoded sites that are predicted to be under diversifying natural selection in the framework region, which seems especially surprising given that germline genes are themselves a product of natural selection. These selection factors change during the process of affinity maturation. Although similar observations have been made by earlier authors as described above, we were able to see these in greater resolution.

We hope that our modeling efforts will improve antibody optimization. Currently, antibody optimization for therapeutic development often relies on systematic mutagenesis approaches, such as comprehensive alanine scanning or saturation mutagenesis of CDR residues, which requires testing thousands of variants. Our DNSM model could significantly streamline this process by allowing researchers to input their lead candidate sequence and receive site-specific selection factors indicating which positions are most amenable to beneficial mutations, focusing experimental efforts on the most promising sites. Additionally, our finding that FWRs contain numerous sites under diversifying selection suggests that antibody engineers should not limit optimization efforts solely to CDRs.

There are many opportunities to improve this model. First, because this model doesn’t know anything about the antigen, it is effectively an average across antigens. Thus, one future direction is fine-tuning using antigen-specific data (Wang et al. 2025). Also, moving forward we will generalize the type of data that can be used for the model, including paired and partial sequences. We have also only trained our models on natural human repertoires, and thus we have not attempted to validate the model’s performance on synthetic or other nonhuman antibodies. However, we plan to fit models for other species in the future. Additionally, our current approach models selection effects only at the amino acid level, but selection also operates at the codon level through effects on RNA stability or expression. This could be incorporated into future models.

On a more technical note, we acknowledge a mathematically unappealing aspect of the model: the model predicts a probability of nonsynonymous substitution as a neutral probability of nonsynonymous mutation times a scaling factor *f*. Because there aren’t any hard limits on the size of *f*, this can lead to probabilities that are greater than one. The classical approach, which formulates the model in terms of rates of a continuous-time Markov chain, does not have this defect. However, as described in the *Model alternatives* section of the appendix, we have not found a way to do this in a tractable way given the complexity of the models used. On a practical note, after a moderate amount of training, the model rarely produces predictions outside the range of probability.

Overall, we believe that harnessing the power of natural language processing in the service of evolutionary modeling will lead to a greater understanding of the biophysical aspects of antibodies. An interesting application would be to use the model for antibody combinatorial library design, by indicating sites that are more amenable to change.

In future iterations of this project, the natural selection model will supply $f_{\;j,a}$, a natural selection factor, for every alternate amino acid *a*. We would then estimate the probability of transitioning to a non-wildtype (non-WT) codon *c* at codon site *j* under the mutation-selection model to be


$$p_{\;j,c}(t,X)f_{\;j,\bar{c}}(\bar{X})$$

where $\bar{c}$ is the amino acid for codon *c* and $\bar{X}$ is the complete amino acid sequence of the parent. The probability of maintaining the same codon will be one minus the collection of these transition probabilities. This would furnish a model that would be suitable for comparing to deep mutational scanning data. Furthermore, we believe that the embedding layer of this model will be useful to suggest ways to improve antibodies (Hie et al. 2024; Chinery et al. 2024) and as an input for regression methods to predict functional properties of antibodies as in (Bachas et al. 2022; Barton et al. 2024) and many other papers. Also, this model may be able to help discriminate between generic antigen-agnostic antibody selection versus antigen-specific changes in a manner analogous to models of natural selection in viruses (Bloom 2017).

## Materials and Methods

### Data

BCR sequence data is processed with partis (Ralph and Matsen 2016a, 2016b, 2019) to cluster into clonal families and infer germlines. Inferred insertions or deletions are reversed, so that all sequences align to the naive sequence without gaps. We select clonal families with at least two productive sequences; a sequence is considered productive if the canonical cysteine and tryptophan codons that flank the CDR3 are in the same frame as the start of the V segment (although they can be mutated), and there are no stop codons.

Tree inference and ancestral sequence reconstruction are performed with K80 substitution model and naive sequence as outgroup, allowing mutation rate heterogeneity across sites using a 4-category FreeRate model using IQTree (Minh et al. 2020).

Once this is done, we have a set of PCPs that correspond to the pairs of parent and child sequences on the edges of the phylogenetic tree (Fig. 1a). We use these PCPs to train the model.

We denote pairs of parent and child sequences as (X,Y), where *X* is the parent sequence and *Y* is the child sequence. We use $\bar{X}$ to denote the amino acid sequence corresponding to *X*.

### Model

A detailed diagram describing the modeling framework appears in supplementary fig. S1, Supplementary Material online.

We take a Poisson model of mutation using rates derived from parent sequences, and these rates will get scaled by a deep natural selection model (DNSM) *f*. Specifically, $f_{\;j}(\bar{X})$ is the selection factor modifying the probability of substitution of the current amino acid at site *j* of amino acid sequence $\bar{X}$.

- If $f_{\;j}(\bar{X})>1$, it means that we are more likely to see an amino acid change at site *j* of $\bar{X}$ than under neutral evolution.
- If $f_{\;j}(\bar{X})<1$, it means that we are less likely to see an amino acid change at site *j* of $\bar{X}$ than under neutral evolution.

Thus this *selection factor* has an interpretation similar to a dN/dS ratio (Yang and Bielawski 2000). However, it is not a ratio of rates so we give it a different name. Instead, the selection factor is a multiplicative modifier *f* of the probability of nonsynonymous mutation relative to a neutral baseline (Fig. 1b).

These definitions are straightforward to understand when we are describing the evolution of a germline-encoded gene, but they also extend to the case when a residue isn’t germline-encoded. For example, if we see a large *f* for an arginine at site *j* of the nontemplated part of the CDR3, it means: if we were to see that arginine appear as part of VDJ recombination (and perhaps also SHM), then selection would favor its modification. As another example, if $f_{j}$ is small for a nongermline-encoded amino acid (i.e. a mutated amino acid) at site *j* of $\bar{X}$ in a region that is encoded by the V gene, the model is predicting that this mutated residue is under purifying natural selection. We also emphasize that the selection factor for a site *j* depends on the whole sequence $\bar{X}$, including the amino acid at site *j*.

To explain in more detail, let’s consider a single PCP, for which we have an estimated branch length of *t*. We use $\lambda_{i}$ to denote the mutation rate at site *i* due to SHM. Recall that the SHM model is fixed.

The neutral SHM probability of a mutation at site *i* of the codon to base *b* is $(1-\exp\;(-\lambda_{i}t))s_{ib}$ where $s_{ib}$ is the probability of substitution to base *b* given that there is a substitution at site *i*. The probability of no mutation at that site is $\exp\;(-\lambda_{i}t)$. By taking the product of such probabilities for the sites of a codon, we get the probability of various codon-level transitions.^1^ For example, if we are considering the probability of the codon formed by nucleotide sites 7, 8, 9 to mutate from AGC to AGG, the probability would be


(1) $$\exp\;(-\lambda_{7}t)\exp\;(-\lambda_{8}t)\left(1-\exp\;(-\lambda_{9}t)\right)s_{9G}.$$

In this way for every codon index *j* we get $p_{\;j,c}(t)$ where *c* are alternate codons (Fig. 1b).

This model formalizes the idea that the rate of a multinucleotide mutation is going to be the rate of those events happening independently. One could imagine using a model (Lucaci et al. 2021) that takes into account the clustered nature of somatic hypermutation (Spisak et al. 2020) but this is not done here.

Our overall goal is to predict the probability of a nonsynonymous substitution at each site for a given parent one-hot-encoded antibody sequence *X* (Fig. 1b). Given the corresponding (parent) amino acid sequence $\bar{X}$, the natural selection model returns $f_{j}$: a nonnegative natural selection factor for each site *j*. This is multiplied by a probability $p_{j}(t)$ of a nonsynonymous mutation at site *j* (defined below) to get the probability of a nonsynonymous mutation at site *j* of $\bar{X}$ after time *t*. This time *t* is free to differ between PCPs, and is jointly optimized with the model *f* (the branch length estimation in the original phylogenetic tree inference is ignored as it does not include either the complex SHM model or the natural selection model). For the purposes of branch length estimation, we estimate the probability of transitioning to non-WT codon *c* at codon site *j* under the model to be^2^


(2) $$m_{\;j,c}(t,X)\;\;=\left\{ \begin{array}{cc} p_{\;j,c}(t,X)f_{j}(\bar{X}) & \text{if}\;c\;\text{codes for a non-WT amino acid}\\ p_{\;j,c}(t,X) & \text{if}\;c\;\text{codes for the WT amino acid}\\ 0 & \text{if}\;c\;\text{codes for a stop codon.} \end{array}\right.$$

The probability of the WT codon is set to be one minus the sum of these probabilities.^3^ Because $m_{\;j,c}(t,X)=p_{\;j,c}(t,X)$ when *c* codes for the WT amino acid, this effectively fixes the selection factors for neutral substitutions to be 1. In the code, we simply ignore the possibility of transitioning to stop codons, which effectively assigns them a transition probability of 0 as above.

For calculating loss of the DNSM, we don’t have to calculate the probability of every alternate codon: we just calculate the probability of a nonsynonymous amino acid substitution. Indeed, the probability of an amino acid substitution at site *j* is the sum of (2) for *c* the set of codons coding for non-WT amino acids, which simplifies to^4^


$$m_{j}(t,X)\;\;=\left(\sum_{c\;\text{codes for non-WT amino acid}}p_{\;j,c}(t,X)\right)f_{j}(\bar{X}).$$

Because the left hand sum is used a lot in neural network training, we precompute and store it^5^ after each round of branch length optimization. We use a binary cross-entropy loss,^6^ which is the negative log likelihood of the observed data under the model.

Note that the $f_{j}$ are not bounded above, and so the likelihoods using the equations above containing $p_{\;j,c}(t,X)f_{j}(\bar{X})$ may not consist of valid probabilities. In practice, probabilities of any particular mutation are small, and even a moderately-trained DNSM will have $f_{j}$’s that are not too large, so we will have these probabilities be less than 1. We handle the rare case where they are greater than one by setting it to a little less than 1 and proceeding with training.

We perform joint optimization of branch length and parameters of the natural selection model. The neutral substitution model parameterizing *p* is inferred using out-of-frame data and fixed. Because we have synonymous and nonsynonymous substitutions, we won’t have an identifiability problem between the branch lengths and the natural selection parameters. For example, a sequence with lots of synonymous substitutions will have a large *t* and will push the *f*’s down. A sequence with lots of nonsynonymous substitutions will have a large *t* and will push the *f*’s up. A sequence without many substitutions at all will just have a small *t*.

### Simulation Validation

To validate parameter recovery and assess the minimum data requirements for reliable model training, we performed a simulation validation experiment. We used a DNSM trained on the Jaffe+Tang dataset as our ground-truth model. Starting with the original Jaffe+Tang naive sequences and phylogenetic trees, we filtered out clonal families containing ambiguities in the naive sequence to ensure clean simulation data. We then refit branch lengths on these trees using the ground-truth model under our mutation-selection framework.

Using the ground-truth model and refit branch lengths, we simulated evolution along the phylogenetic trees by generating PCPs incrementally down each tree, where the child sequence from one edge served as the parent sequence for the subsequent edge. This process generates synthetic sequences that reflect the evolutionary dynamics captured by the ground-truth model while preserving the original tree topologies and naive sequences.

The simulated data were filtered using identical criteria to the real data: we excluded PCPs with identical parent and child sequences as well as naive branches. By design, our simulation process cannot generate stop codons, ensuring all simulated sequences remain in-frame.

We then retrained new DNSM models using varying amounts of the simulated PCP data (from 50,000 to over 500,000 PCPs) to assess how training data volume affects parameter recovery. To evaluate how well these retrained models recovered the ground-truth parameters, we compared predicted selection factors between the retrained models and the ground-truth model on real Rodriguez sequences.

This simulation design isolates the effect of model parameterization from other sources of variation. By using identical tree structures and naive sequences between the ground-truth and retrained models, any differences in performance can be attributed solely to the accuracy of parameter recovery rather than differences in the underlying evolutionary scenarios or data structure. Thus we believe that this approach provides the cleanest assessment of whether our training data volume is sufficient for reliable parameter estimation.

### Wiggle Activation Function

We wished to use a pre-output function such as softplus that smoothly ensures that outputs are non-negative, but has slower-than-linear growth on the positive side because selection factors should not become especially large. Therefore we use a function, which we call the “wiggle” function, to help the selection factors stay within a reasonable range.

This function, after exponentation, is defined as


$$\exp\;(\text{wiggle}(x,\beta ))=\left\{ \begin{array}{cc} e^{\beta (x-1)} & \text{if}\;x<1,\\ x^{\beta } & \text{if}\;x\geq 1. \end{array}\right.$$

We use β=0.3 in our study (supplementary fig. S14, Supplementary Material online). A trainable *β* was tested but did not make a difference in performance.

### Relative Solvent Accessibility

Relevant solvent accessibility values are computed with the DSSP program (Kabsch and Sander 1983; Touw et al. 2014) via the Biopython package. The program takes a protein PDB file of atomic coordinates as input. We use the acc_array=’Wilke’ reference values for accessible surface area (Tien et al. 2013).

### Model Implementation, Training, and Evaluation

This model is implemented in PyTorch (Paszke et al. 2019). Models are trained using the RMSprop optimizer, with 4 cycles of branch length optimization then neural network optimization.

We use the following software: biopython (Cock et al. 2009), logomaker (Tareen and Kinney 2020). matplotlib (Hunter 2007), pandas (McKinney 2010), pytest (Krekel et al. 2004), PyTorch (Paszke et al. 2019), seaborn (Waskom 2021), and snakemake (Mölder et al. 2021).

GPT-4, GitHub Copilot, and Claude were used to draft modeling and plotting code for this project.

## Supplementary Material

msaf186_Supplementary_Data

## Data Availability

Models and inference code can be found at https://github.com/matsengrp/netam. The reproducible experiments, including trained model weights, are available at https://github.com/matsengrp/dnsm-experiments-mbe. Figs. 2, 5, 8, supplementary figs. S2, S3, S12, and S13, Supplementary Material online are made in dnsm_oe.ipynb. Fig. 3 is made in dnsm.ipynb. Figs. 4, supplementary figs. S5, and S6, Supplementary Material online are made in dnsm_simulation_panel_eval.ipynb. Figs. 6, 7, and supplementary figs. S9, Supplementary Material online are made in along_tree.ipynb. supplementary figs. S8, S10, and S11, Supplementary Material online are made in surprising_sites.ipynb. Fig. 9 and supplementary table S3, Supplementary Material online are made in dnsm_vs_asa.ipynb. supplementary fig. S14, Supplementary Material online is made in wiggle.ipynb. These notebooks can be found in notebooks/dnsm_paper directory of the experiments repository. All preprocessed data is available on Zenodo at https://doi.org/10.5281/zenodo.15931790.

## Footnotes

1
codon_probs_of_mutation_matrices in molevol.py.

2 Selection factors are computed in _build_selection_matrix_from_parent in dnsm.py, which is needed to define the objective function for branch length optimization defined in mutsel_log_pcp_probability_of in molevol.py.

3
build_codon_mutsel in molevol.py.

4
DNSMBurrito.predictions_of_pair in dnsm.py.

5
DNSMDataset.update_neutral_probs in dnsm.py

6 last line of DNSMBurrito.loss_of_batch in dnsm.py.
