# Supplementary material for: A Sitewise Model of Natural Selection on Individual Antibodies via a Transformer–Encoder
Source: Mol Biol Evol. 2025 Aug 5;42(8):msaf186. doi: 10.1093/molbev/msaf186 (PMC12375951; doi:10.1093/molbev/msaf186)
Supplement: msaf186_Supplementary_Data [file msaf186_supplementary_data.pdf]

Supplementary Materials

Supplementary figures and tables

purpose	name	samples	clonal families	PCPs	median mutations
train	`Tang50k`	21	25,410	51,759	3
train	`Tang`	21	43,878	517,605	3
train	`Jaffe+Tang`	25	97,626	743,278	4
train	`SimJaffe+Tang`	25	85,866	575,760	4
test	`Rodriguez`	50	3,065	21,587	6

Table S1: Data used in this paper. `Tang` data is from (Tang et al., 2020; Vergani et al., 2017) and was sequenced using the methods of Vergani et al. (2017); `Tang50k` is a subset of that data. `Jaffe+Tang` is a combination of the `Tang` data and the `Jaffe` data from (Jaffe et al., 2022) sequenced using 10X. `SimJaffe+Tang` is simulated data designed to mimic `Jaffe+Tang`. `Rodriguez` data is the 5′ RACE data from (Rodriguez et al., 2023) and is used only for testing. The "samples" column is the number of individual samples in the dataset; in these datasets, each sample is from a distinct individual. "Clonal families" is the number of clonal families in the dataset. "PCPs" is the number of parent-child pairs in the dataset. "Median mutations" is the median number of mutations per PCP in the dataset.

Label	Heads	Model dim per head	Layers	Parameters
1	-	-	-	1
13K	4	4	3	13,473
77K	4	8	5	76,929
1.2M	8	16	5	1,192,449
4.7M	8	32	5	4,744,193

Table S2: Models used in this analysis. Model labeled "1" is a baseline model with a single constant selection factor. The others are transformer-encoder models as described in the text.

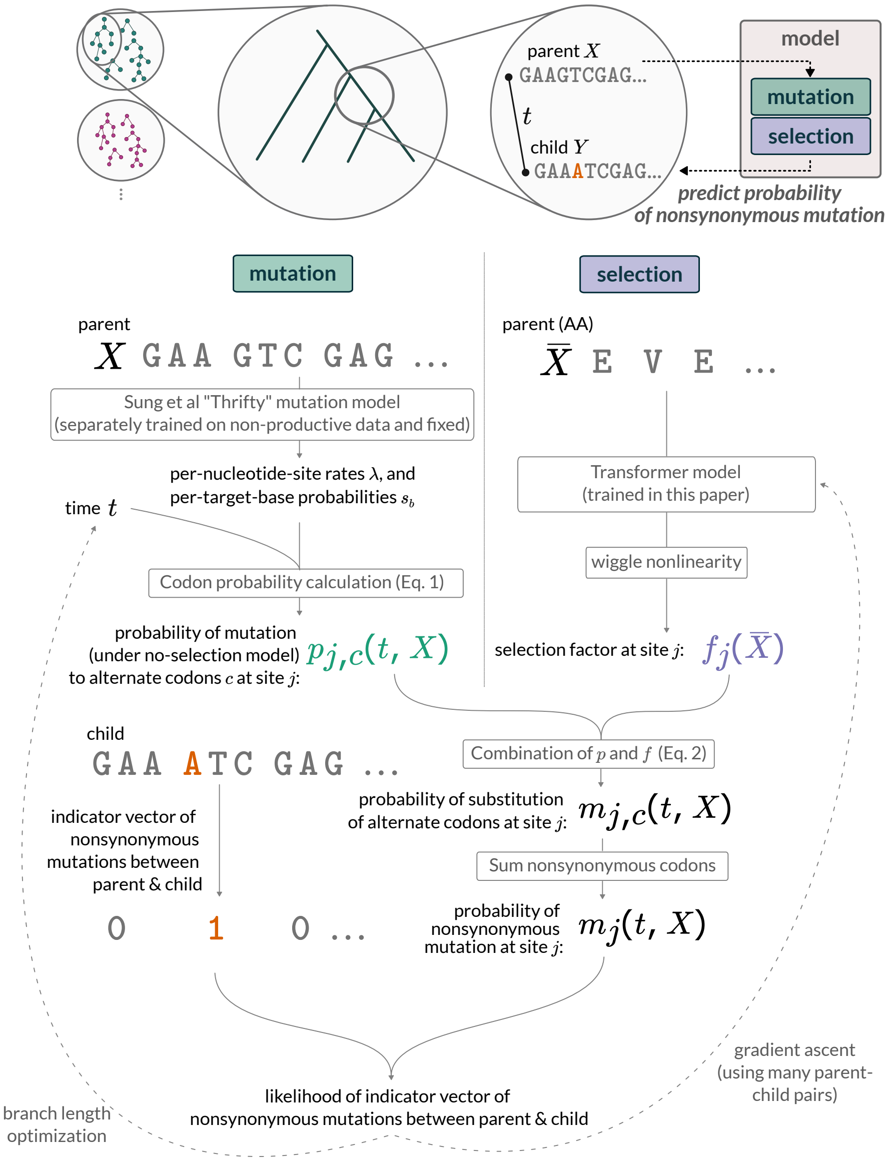

Figure S1: The model is trained to predict the probability of nonsynonymous mutation between parent and child sequences reconstructed from B cell receptor clonal families. It is divided into mutation and selection components. Mutation: given a parent sequence X, the probability of mutation to alternate codons after time t is calculated using a model of SHM (Sung et al., 2025) and then aggregated into codons as in (1) to obtain $p_{j,c}(t, X)$. Selection: given an amino acid translation $\bar{X}$ of the parent, the transformer-encoder gives per-site selection factors $f_{j,c}(\bar{X})$. These are then combined (2) and summed to give the probability of nonsynonymous mutation at every site. This gives a likelihood for a parent-child pair. The algorithm maximizes the likelihood across branch lengths t for each parent-child pair as well as across the parameters of the transformer model for all parent-child pairs in the dataset (dashed lines).

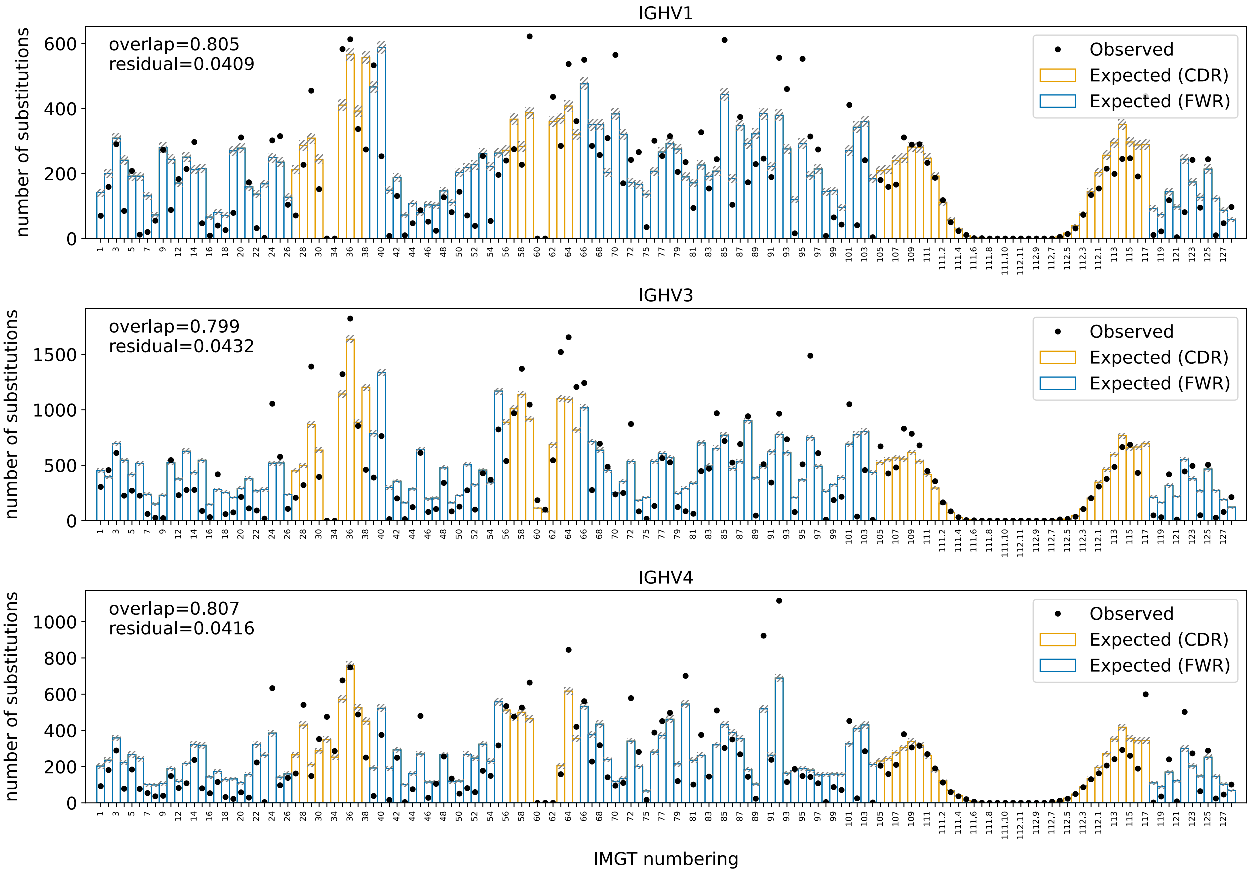

Figure S2: Model fit for a baseline model with a single selection factor. Compare Figure 2.

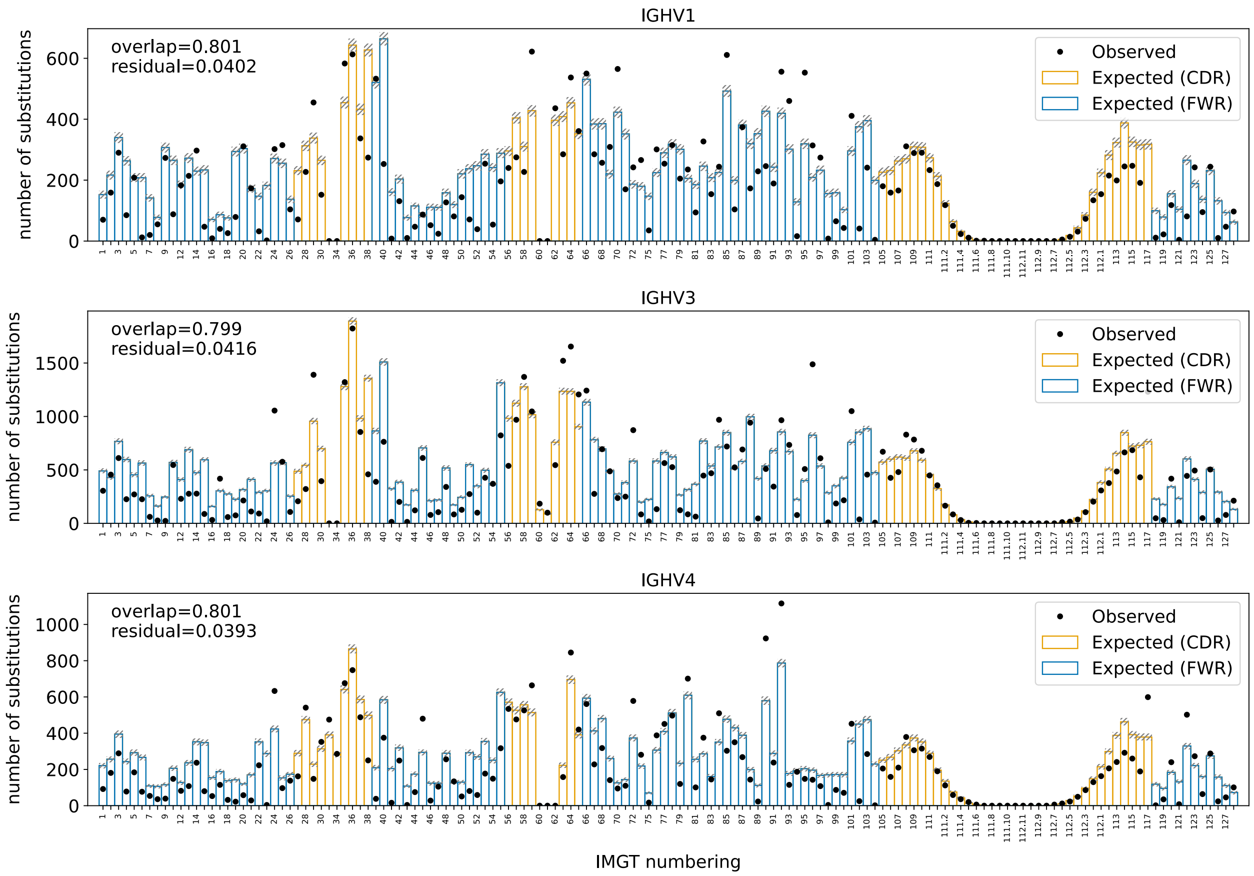

Figure S3: Model fit for a baseline model with a no selection factor. Compare Figure 2.

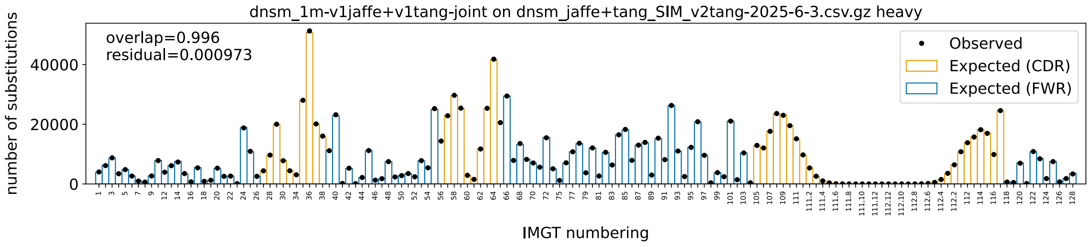

Figure S4: Simulated substitutions closely match predictions under the model used for simulation, showing that the simulation procedure is faithfully recapitulating the inferential model.

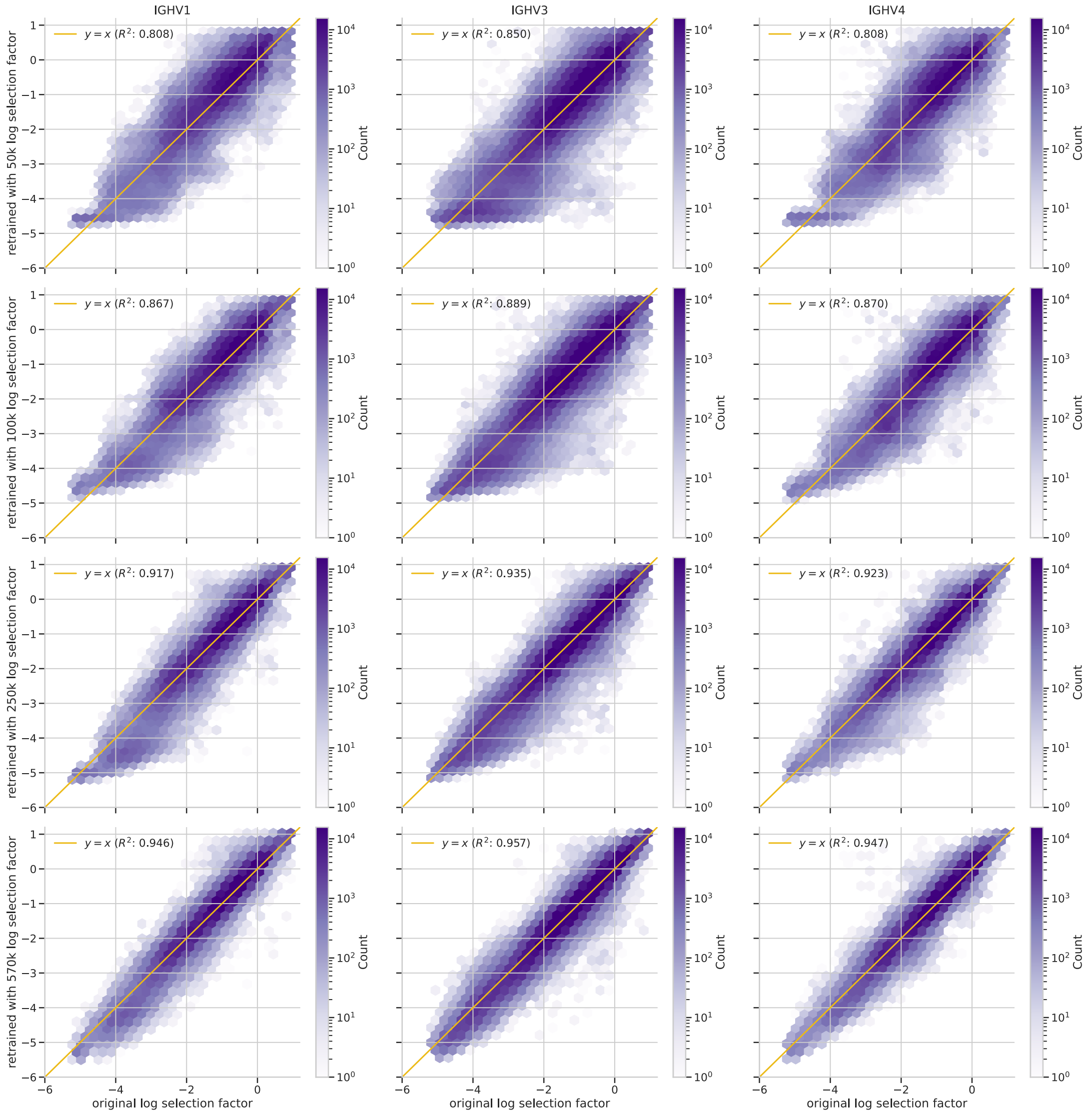

Figure S5: Simulation validation as in Figure 4 but for additional subsample sizes.

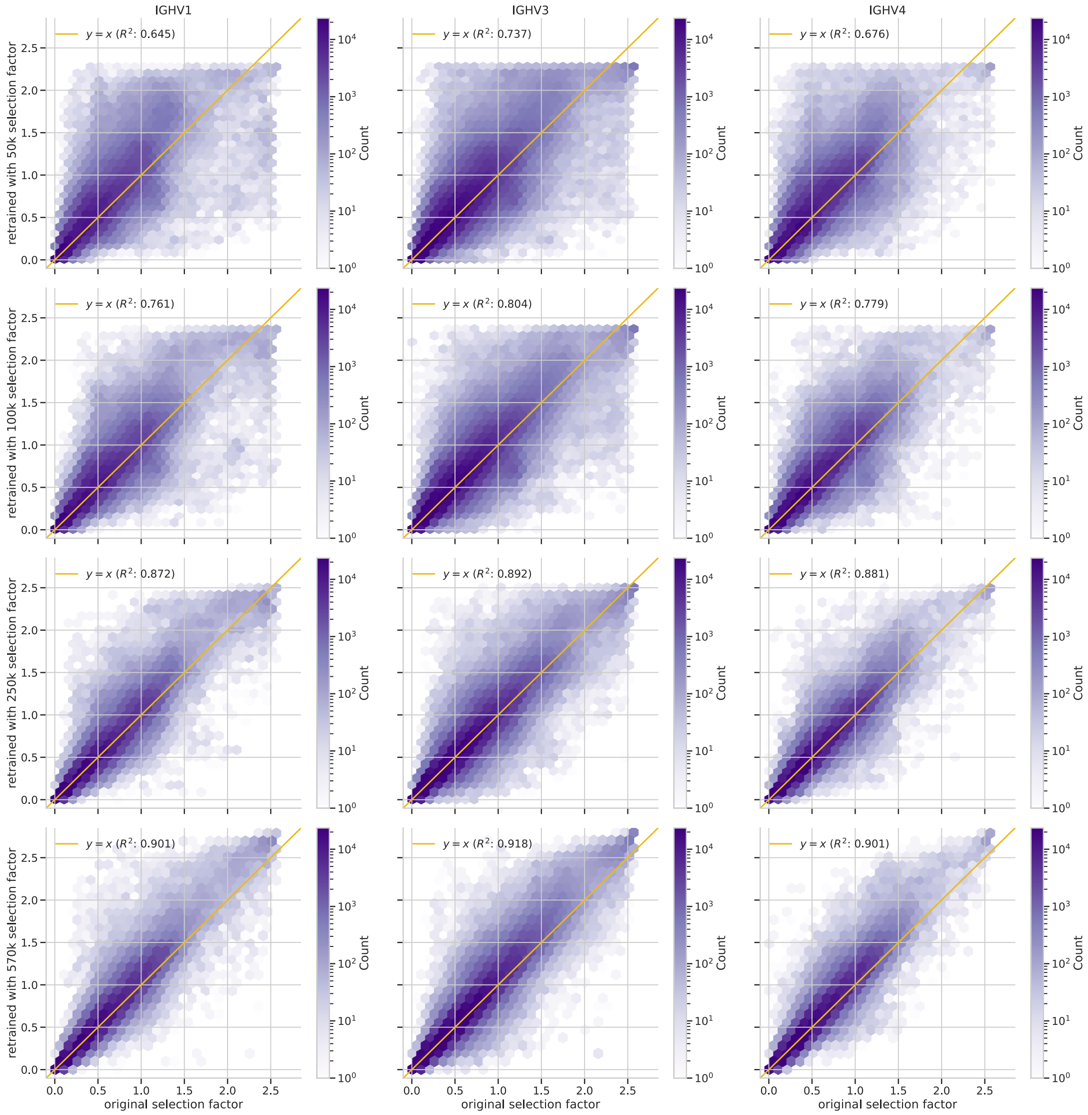

Figure S6: Simulation validation as in Figure S5 but in linear rather than log scale.

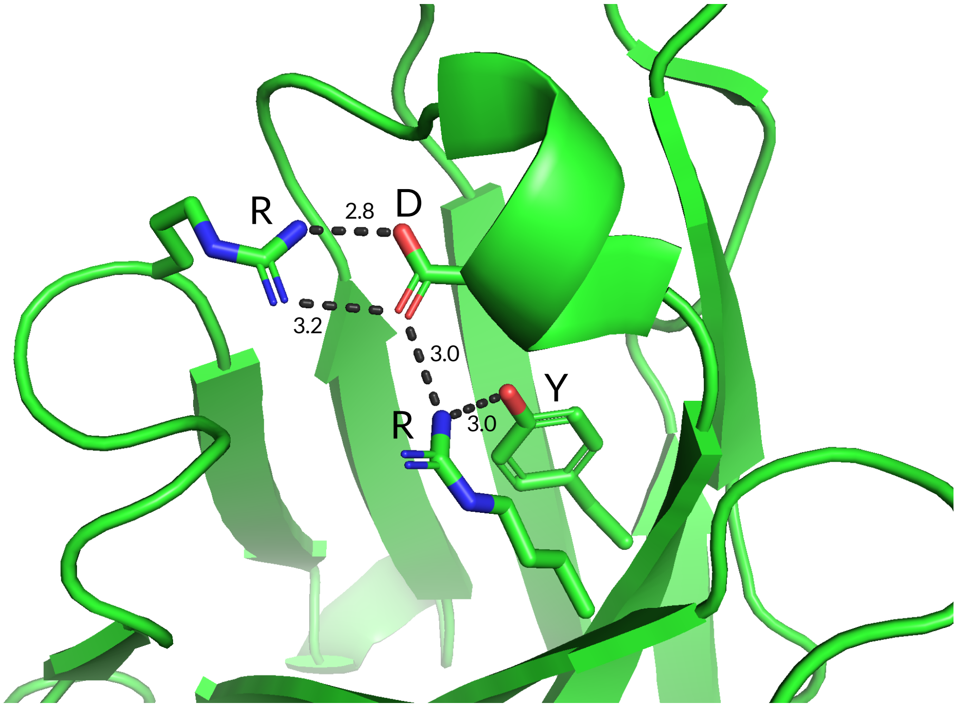

Figure S7: The RRDY motif, which we observe to be under strong purifying selection, forms electrostatic interactions at the base of the variable region.

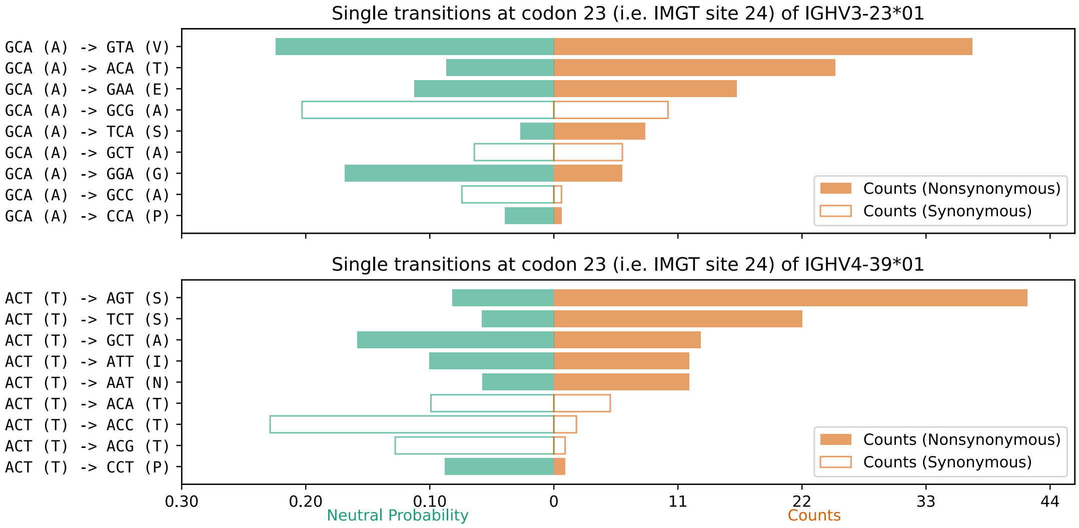

Figure S8: Nonsynonymous counts dominate for single-codon transitions at codon 23 (i.e. IMGT site 24) of the most commonly used V3 and V4 genes in the `Rodriguez` data set. Orange right hand bars show the counts for each of the nine possible single-nucleotide codon mutations for IGHV3-23*01 and IGHV4-39*01 for PCPs in the `Rodriguez` data set. This dominance is not driven by the neutral mutation probabilities (green left hand bars). These neutral probabilities are the normalized probability of these nine possible single-nucleotide codon probabilities at that site, averaged across all sequences in the corresponding subset of the data. A visualization of the selection factors in a structural context can be seen at `https://matsen.group/dnsm-viz/v1/?pdbid=3b2v`.

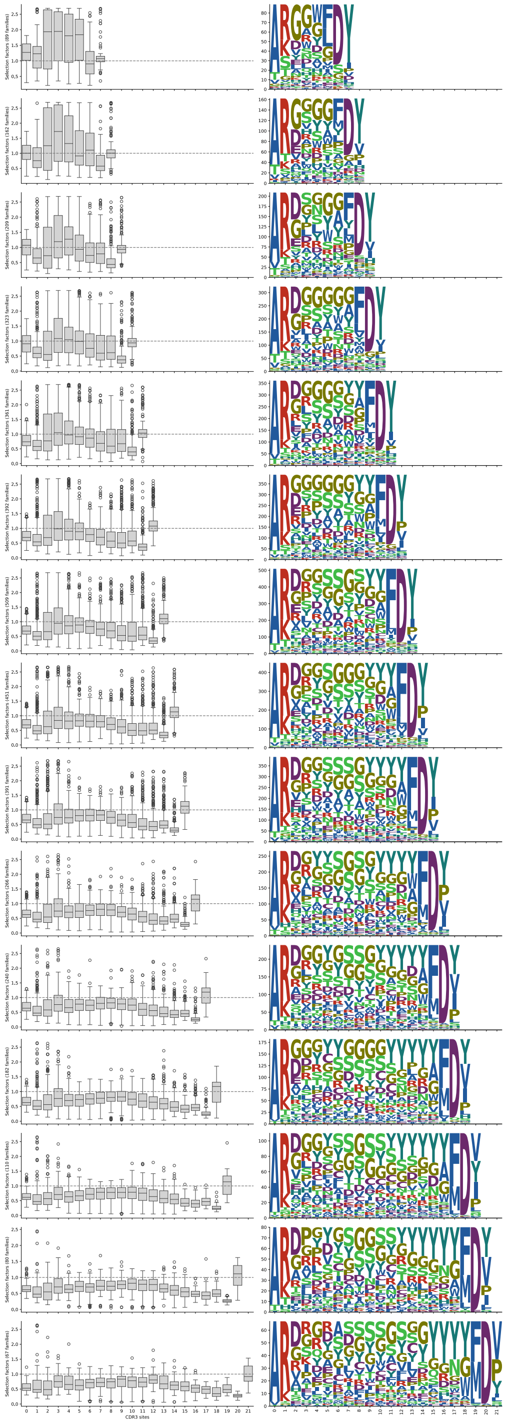

Figure S9: Distribution of ancestral selection factors per site for CDR3s of various lengths, as well as the logo plots for those ancestral sequences. The plot in Figure 7 takes the median of these boxplots, and shows position along the CDR3 normalized by sequence length, with the row position in this plot shown as the x axis in that plot.

IMGT site	region	amino acid	median RSA	median selection factor
118	FR4	W	0.351	0.0798
16	FR1	G	0.529	0.101
9	FR1	G	0.358	0.104
52	FR2	W	0.316	0.111
8	FR1	G	0.394	0.125
7	FR1	S	0.368	0.141
18	FR1	S	0.401	0.164
46	FR2	P	0.604	0.166
15	FR1	P	0.403	0.185
27	CDR1	G	0.548	0.187

Table S3: The 10 IMGT sites with a median RSA of more than 0.3 that are predicted to be under the strongest purifying selection for antibody sequences in the SAbDAb. The "amino acid" column labels the most frequent amino acid at that site.

IMGT site	region	amino acid	median RSA	median selection factor
87	FR3	A	0	0.74
78	FR3	I	0.0305	0.616
105	CDR3	A	0	0.52
76	FR3	F	0.0208	0.517
91	FR3	M	0	0.491

Table S4: The 5 IMGT sites with a median RSA of less than 0.05 that are predicted to be under the weakest purifying selection for antibody sequences in the SAbDAb. The "amino acid" column labels the most frequent amino acid at that site.

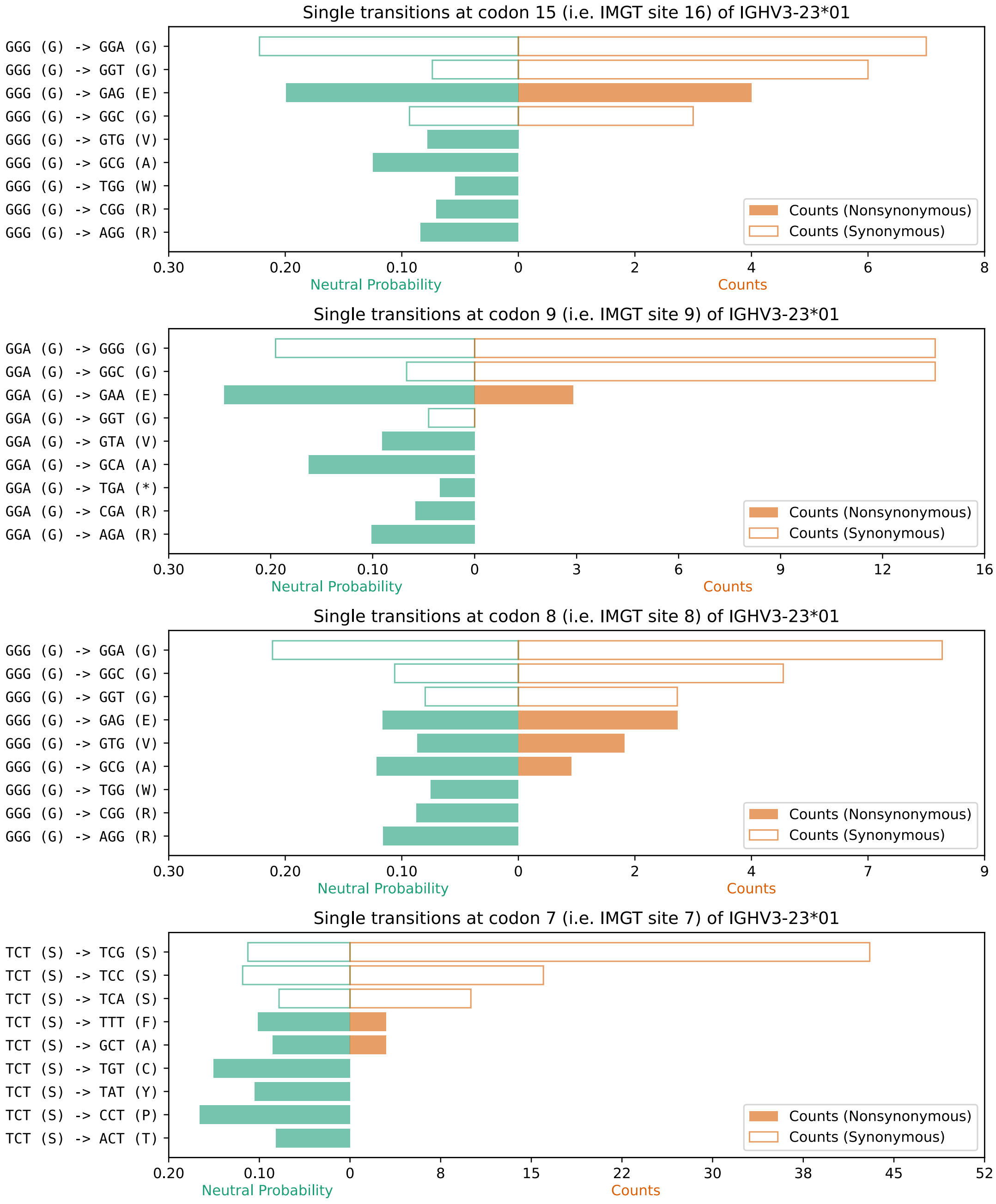

Figure S10: Purifying selection at three exposed residues (RSA > 0.3, see Table S3) with low selection factors for the most commonly used V3 gene in the `Rodriguez` data set; counting and presentation as in Figure S8. A visualization of the selection factors in a structural context can be seen at `https://matsen.group/dnsm-viz/v1/?pdbid=5i19`.

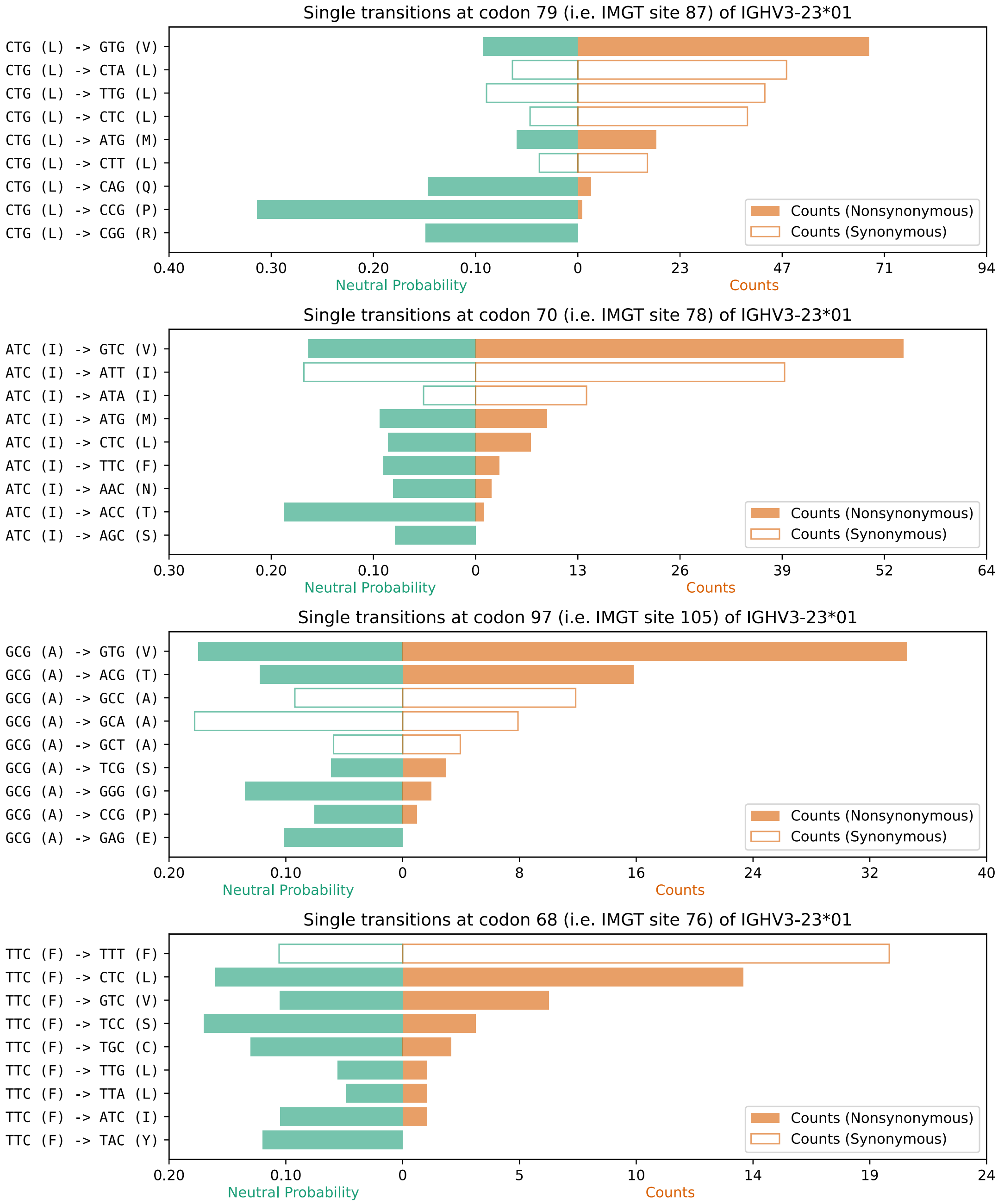

Figure S11: Evidence that some nonsynonymous substitutions are tolerated at three amino acid positions that are quite buried (RSA < 0.05, see Table S4), but have higher selection factors than other buried sites, for the most commonly used V3 gene in the `Rodriguez` data set. Counting and presentation as in Figure S8. Note that L was most common for IGHV3-23*10 for IMGT site 87 in this dataset (top panel), however A was most common in the SAbDAb (Table S4). A visualization of the selection factors in a structural context can be seen at `https://matsen.group/dnsm-viz/v1/?pdbid=5i19`.

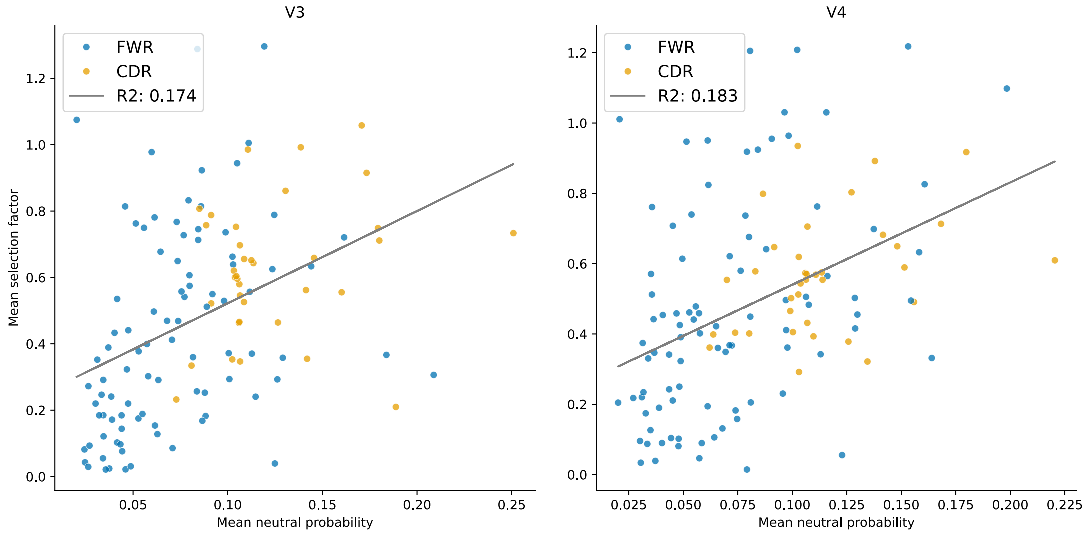

Figure S12: Scatter plot of mutability vs selection, aggregated across sequences using the V3 and V4 gene families.

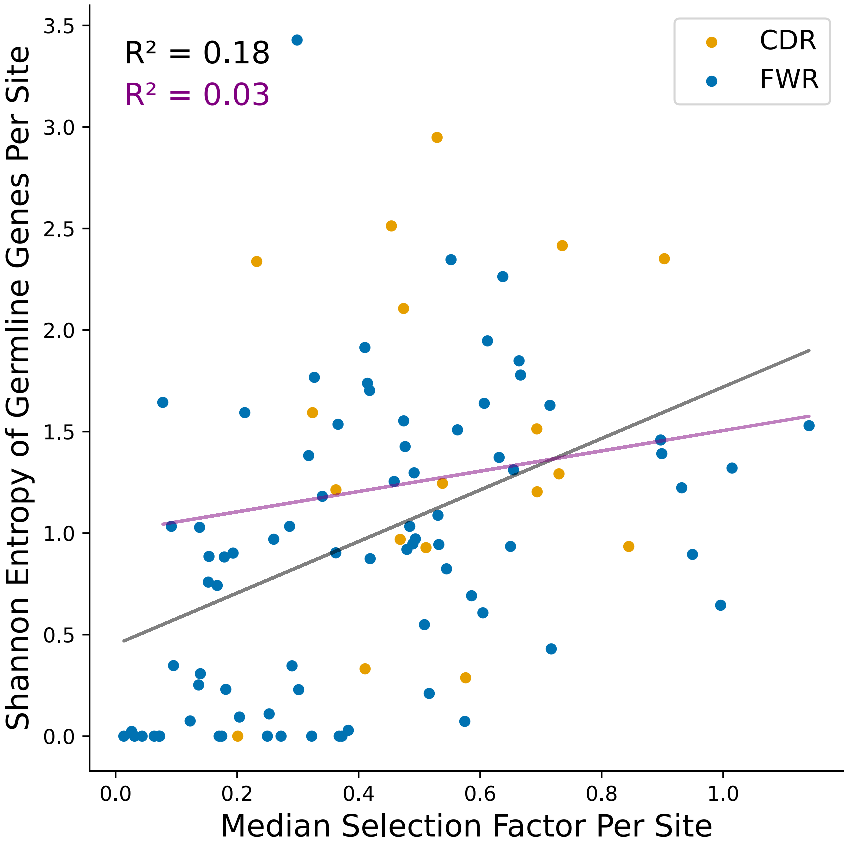

Figure S13: Per-site scatter of the median selection factor versus the entropy of the germline genes, using the IMGT numbering. Regression line and R^2 for all data (black) and for sites with entropy greater than 0.2 (purple).

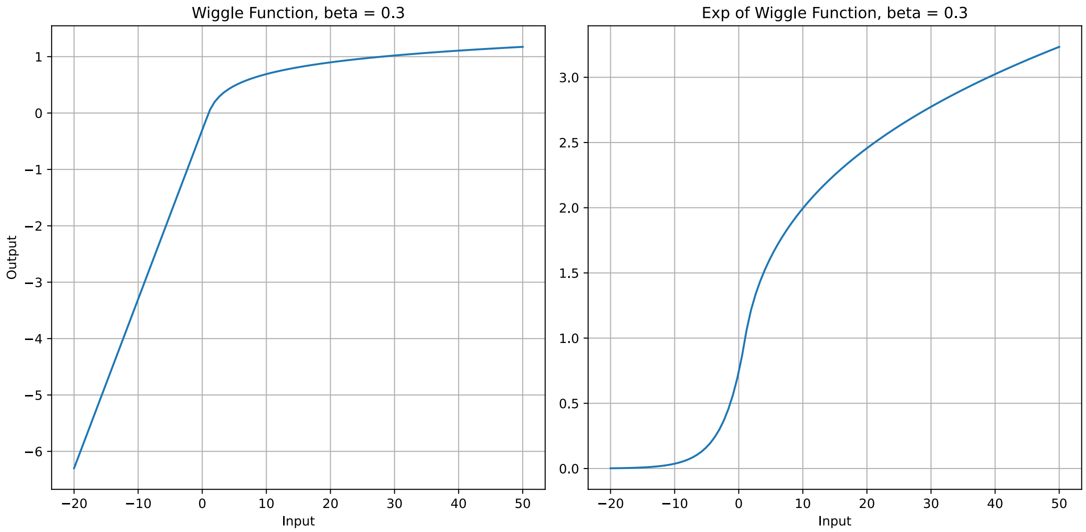

Figure S14: The wiggle activation function before and after exponentiation.

Model alternatives

In this work, we multiply the probability of a nonsynonymous mutation by a multiplicative scaling factor f that we call a selection factor (2). Although this formulation has proven useful in other immune receptor settings (Elhanati et al., 2014), it is an atypical choice for evolutionary modeling. Here we explain why we chose this formulation.

The most common way to numerically describe natural selection is as a rate multiplier $\omega = d_N/d_S$ in a continuous-time Markov chain model (Yang and Bielawski, 2000). In contrast, our SHM models (Sung et al., 2025) follow the rest of the SHM literature (starting with the highly influential (Yaari et al., 2013)) by conditioning on a stretch of sequence around the focal base. This wide conditioning is at odds with the site-independent assumption required to make a CTMC tractable. Thus, we will calculate mutation rates conditioned on the parent sequence and consider the waiting time until a mutation as being exponentially distributed with those rates.

One might still wish to build something like a Poisson version of a codon model, in which one would calculate rates of substitution to each of the codons, rather than probabilities. However, to compute the probability of getting multiple mutations, we have to work in probability land rather than rate land, because the maximum of two independently exponentially-distributed events is not exponentially distributed: the CDF of getting two events, with rates λ_1 and λ_2, before time t is $(1 - \exp(-\lambda_1 t))(1 - \exp(-\lambda_2 t))$. As an intuitive example, imagine that we are considering two sites, in which the first site has a high rate of mutation, and the second site has a low rate of mutation. Consider the probability of mutation to a codon in which the first site is not mutated, but the second site is mutated. This cannot be approximated as a constant rate through time, because the probability of this case will go down as time gets large, driven by the probability of not having a mutation in the first site. Nevertheless, this may be an appropriate approximation for short time scales, and we may investigate it in the future.

From a practical perspective, the current formulation is convenient because the model log likelihoods are expressed as the sum of the mutation and the selection log likelihoods. This is efficient for model fitting via backpropagation.
